# Solutions and Integrated Strategies for the Control and Mitigation of Plastic and Microplastic Pollution

**DOI:** 10.3390/ijerph16132411

**Published:** 2019-07-07

**Authors:** Joana C. Prata, Ana L. Patrício Silva, João P. da Costa, Catherine Mouneyrac, Tony R. Walker, Armando C. Duarte, Teresa Rocha-Santos

**Affiliations:** 1 Centre for Environmental and Marine Studies (CESAM) & Department of Chemistry, University of Aveiro, 3810-193 Aveiro, Portugal; 2 Centre for Environmental and Marine Studies (CESAM) & Department of Biology, University of Aveiro, 3810-193 Aveiro, Portugal; 3 Mer Molécules Sante (MMS), Université Catholique de l’Ouest, 3 place André Leroy, BP10808, 49008 Angers CEDEX 01, France; 4 School for Resource and Environmental Studies, Dalhousie University, Halifax, NS B3H 4R2, Canada

**Keywords:** marine litter, single-use plastics, waste treatment, waste-to-energy, feed-stock, bioplastics

## Abstract

Plastic pollution is generated by the unsustainable use and disposal of plastic products in modern society, threatening economies, ecosystems, and human health. Current clean-up strategies have attempted to mitigate the negative effects of plastic pollution but are unable to compete with increasing quantities of plastic entering the environment. Thus, reducing inputs of plastic to the environment must be prioritized through a global multidisciplinary approach. Mismanaged waste is a major land-based source of plastic pollution that can be reduced through improvements in the life-cycle of plastics, especially in production, consumption, and disposal, through an Integrated Waste Management System. In this review paper, we discuss current practices to improve life cycle and waste management of plastics that can be implemented to reduce health and environmental impacts of plastics and reduce plastics pollution. Ten recommendations for stakeholders to reduce plastic pollution include (1) regulation of production and consumption; (2) eco-design; (3) increasing the demand for recycled plastics; (4) reducing the use of plastics; (5) use of renewable energy for recycling; (6) extended producer responsibility over waste; (7) improvements in waste collection systems; (8) prioritization of recycling; (9) use of bio-based and biodegradable plastics; and (10) improvement in recyclability of e-waste.

## 1. Introduction

Plastic accumulation in the environment is increasing due to low degradation rates coupled with unsustainable use and disposal [1]. In 2016, global production of plastics reached 322 million metric tons, with a large portion (39.9% in Europe) being used in packaging [2]. An estimated 4.8 to 12.7 million metric tons of mismanaged plastic waste entered the oceans worldwide in 2010 [3]. When exposed to environmental conditions, plastics fragment via photo and thermo-oxidative degradation producing particles <5 mm, known as secondary microplastics [4]. Polyethylene (PE), polypropylene (PP), and polystyrene (PS) are the most common plastics found in the oceans, the most commonly produced plastics worldwide [4]. Plastic behavior in the environment is influenced by its properties, such as density, but also by adsorption of organisms (biofouling) and chemicals to its surface, which also contribute to toxicity [1]. Up to 102,000 microplastics m^−3^ have been found in a Swedish harbor [5]. High concentrations of microplastics are usually found in coastal areas near industrial facilities, ports and large metropolitan areas [6]. In the open ocean, gyres produced by wind and currents lead to accumulation of plastic marine debris [1]. Plastics of all sizes have potential to cause social and economic losses (e.g., damage to fishing equipment; loss in tourism revenue) [7] and impose important threats to natural ecosystems and human health [8,9,10,11,12]. Plastic pollution is thought to be a planetary boundary threat, as it is irreversible (low degradation, impossibility to recover all plastics), at planetary-scale, and likely to disrupt Earth system processes, either by having negative effects on ecosystems or by altering the physicochemical properties of the environment [13]. The life-cycle of plastics and plastic pollution contributes directly to climate change and has negative impacts on biodiversity loss [14].

Clean-up activities have been proposed as mitigation strategies, as well as tools for awareness and citizen science [15]. However, removing all plastics from the environment seems impossible due to widespread environmental pollution with plastic particles of all sizes [16]. Although, clean-up actions help to reduce pressure of plastic pollution on ecosystems, they are insufficient to control this pervasive problem. Conversely, availability of science-based solutions and technology lack integration and implementation across a number of sectors, and by stakeholders worldwide. Long-lasting solutions require multidisciplinary approaches and international cooperation, since plastic pollution affects neighboring countries and international waters. These include national and international governance [17,18,19,20,21,22,23], reducing release of plastics in wastewater treatment plants [24,25,26,27,28], consumer education and awareness [29,30], and improvements in life-cycle and end-of-life management.

**Figure 1:**
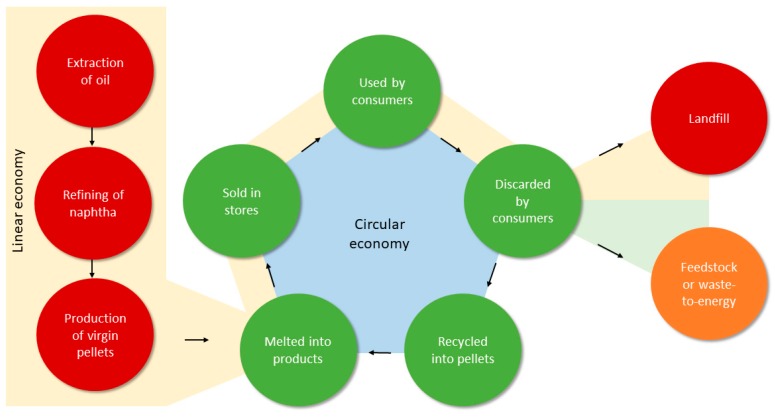
Life-cycle of plastics in a linear economy (cradle-to-grave), from the production of virgin plastic from oil to landfill or recovery (feedstock or waste-to-energy), and circular economy (cradle-to-cradle), where existing plastics are recycled into new plastic products. Feedstock and waste-to-energy are preferred to landfilling, as they allow the recovery of chemical components or energy.

Reducing plastic litter inputs by source-reduction and waste management is one of the most valuable solutions to restore the oceans [3,16,31,32,33]. An integrated waste management system, focused on the four R’s hierarchy (reduce, reuse, recycle, recover) and improving the life-cycle of plastics (Figure 1), is important to reduce energy and resources consumptions, avoid harmful emissions [34,35], and reduce quantities of mismanaged plastic waste reaching the oceans. The objective of this review is to discuss current strategies to improve sustainability of plastics during the entire life-cycle, including in waste management, providing stakeholders with practical recommendations. Strategies have been organized in three sections: (1) production of plastics dealing with improvements at an industrial level, including usefulness of life cycle assessments; (2) consumption of plastics products by reducing their appeal to consumers, especially through education; and (3) waste management advocating for the recycling and recovery of plastic waste. Key findings from this review were developed into recommendations for stakeholders to improve the management of plastics. Recommendation presented herein will contribute to the implementation of a circular economy (from cradle-to-cradle) and reduction of mismanaged plastic waste entering the oceans [16].

## 2. Improving Production Efficiency of Plastic Products

At production level, the use of plastics can be reduced by (a) using alternative (e.g., glass), recycled, or biodegradable materials; (b) improving the design to reduce the amount of plastic used, extend product life, allow repair and reuse, and improve recyclability by limiting the number of polymers, additives, and mixtures; and (c) banning certain types of single-use plastics [1,21,23,36,37]. An example of improved design is making caps inseparable from plastics bottles to increase their correct disposal [16], however this could impair recyclability due to the presence of two polymer types. There is a demand for improvements in design, which also benefit companies by reducing requirements for raw materials, and for viable alternatives, which are still limited. Recycled are more expensive than virgin plastics, however they are beneficial at an environmental and societal level [38] and thus should be encouraged by voluntary (as a marketing strategy) or mandatory incorporation of percentage of recycled materials (e.g., 10% of weight), which cannot be too high due to losses in each recycling cycle [39].

Even though plastic is rarely substituted in some products (e.g., food packaging), full bans of other single-use plastics are beneficial [40], such as in cotton buds and microbeads added to cosmetics, that often comprise marine litter and can be substituted by biodegradable materials [21,23,41,42,43]. Indeed, microbeads have been banned in Canada, New Zealand, United States, and United Kingdom [43]. However, bans of single-use products should consider their impact on public health. For instance, medical equipment and packaging of perishable foodstuff (e.g., fresh meat) should not be banned but improved in design. In Taiwan, plastic bags had to be partially reintroduced due to concerns with food safety, reverting a full ban on plastic bags [44]. Rochman and Browne [45] also believe that problematic plastics, such as polystyrene, polyurethane, polycarbonate, and polyvinyl chloride, need to be classified as hazardous and substituted with recyclable and safer alternatives, while industries should verify the safety of their plastic products.

Regarding waste, companies should aim at reducing its production and recycling during manufacturing through a combination of voluntary and mandatory measures. For example, loss of pre-production plastic pellets can be minimized by voluntary (e.g., Operation Clean Sweep) or mandatory (e.g., National Pollutant Discharge Elimination System in California) strategies, such as installing meshes in drains. Furthermore, corporations should also be accountable for the waste produced by their products in an Extended Producer Responsibility (EPR), usually by paying a recycling fee or through deposit schemes, leading to reductions in waste and increased support for recycling.

All measures can be applied voluntarily, or through mandatory actions which include (a) imposing ecological requirements, labelling of harmful substances, or taxes varying with product recyclability; (b) giving fiscal incentives or subsidies to recycling companies or companies using recycled materials, giving concessions to environmental friendly activities; (c) attributing awards (e.g., financing marine litter collection by fishermen) and favor the purchasing of recycled goods [36,40,46,47,48]. However, mandatory actions require surveillance to ensure no free riders [48]. For example, Canada already aims at the inclusion of at least 50% of recycled plastics in products by 2030 [49] and has announced phase out of single-use plastics by 2021 [50]. The European Union is cataloguing [51] and creating lists of recommended plastic additives [52].

### Life Cycle Assessment as a Tool to Improve Production

Life cycle assessment (LCA) is a tool to assess environmental impact of a product or process from-cradle-to-grave, limited to a specific case study, functional unit, boundaries, and environmental indicators [53,54], providing an integrated view that helps producers find the most suitable eco-friendly alternative. In several LCA of plastics and alternative materials, end-of-life strategies seem to have smaller environmental impacts when compared to eco-design [55], washing of reusable tableware [56], and food loss caused by inefficient packaging [57,58]. Suggested eco-design improvements include (a) packaging of larger sizes, lower weights, with increased reusability and recyclability; (b) use of lower-energy intensive materials; and (c) eco-friendly means of transportations and efficient shipping configurations [55,58,59]. Nonetheless, increasing recycling rates could significantly decrease environmental impacts. For example, increasing recycling in polyethylene terephthalate (PET) bottles by 25–50% would decrease 5–230% in all environmental impacts [60], whereas a 5% increase in recycling of plastic packaging of legumes would reduce 7% of the global warming potential [55].

End-of-life and energy source can determine if a material is environmentally favorable: plastics outperform glass and aluminum only in jurisdictions with low recycling rates and using non-renewable energy sources [55,59,60,61]. Conversely, these materials outperform plastics in closed-loop systems due to the degradation of plastics during recycling [54,62], such is the case of aluminum bottles [60], glass bottles for olive oil [61], and recycled paper egg packaging in Greece [62]. When considering reusable products, glass cups become less harmful than disposable PS foam cups after 400 servings [63], reusable polycarbonate (PC) cups in small events (but at higher cost) than disposable plastic cups [64], and glass food containers after a lifetime 1.3–3.5 longer than plastic food containers [56]. Thus, LCA for services or products is recommended to support eco-design, and should take into consideration energy sources, end-of-life (including the impacts of mismanaged waste), packaging food loss, and impacts on human health.

## 3. Reducing the Consumption of Plastic

After efficient production, reducing the consumption of wasteful products is beneficial, but sometimes hard to achieve due to food safety and lack of convenience [65]. Nonetheless, avoiding unnecessary packaging (e.g., double-packaging) or choosing eco-friendlier alternatives is still possible. Increasing awareness on environmental impacts of consumer choices through formal (i.e., in schools) or informal (e.g., news, clean-ups) education is a long-term strategy to reduce consumption of plastics, for instance, leading to the choice of microbeads-free alternatives, which could be aided by reliable labelling [1,66,67,68]. An increasing demand for plastic-free products would force companies to re-design their products [33], but alternatives are lacking and require strategies favorable to their development.

Voluntary actions by industries, known as corporate social responsibility (CSR), can be complemented by command and control policies [69,70], including regulation of consumption (e.g., fees), restriction in advertisements, and banning single-use products. Even though consumers support these measures, as shown by the overwhelming citizen support (94%) for the intervention of the European Union on marine litter [71], the same is not always true for manufacturers and retailers, such as the complaint of the violation of free movement of goods by the European packaging manufacturers (Pack2Go) when France banned single-use plastic cutlery [72,73]. Conversely, the intended reduction of lightweight plastic carrier bags in Europe [74], aimed at reducing the yearly loss of 8 billion plastic bags to the oceans [75], translated in some countries into fees (0.10–0.15 €) on previously free plastic bags leading to a reduction in consumption of 74% in Portugal [76] and 90% in Ireland—a measure only criticized by the increase in trash bag sales [77]. Thus, support for government measures may vary between consumers and retailers, requiring thoughtful ponderation.

### Education and Awareness

Education is a powerful tool in the fight against (micro)plastic pollution [78], as demonstrated by the higher amounts of marine litter recovered from beached frequented by citizens of low literacy in Brazil [67] and by the refusal of microbeads products by citizens subjected to awareness campaigns [66]. However, information on (micro)plastic pollution was limited until recently, with 73% of Chilean students not recognizing the problem of microplastics [79]. However, there is a trend for increasing interest in this environmental problem supported by (a) free massive open online courses (e.g., MOOC on Marine Litter) or lectures and activities (e.g., TechWild; The Oceans Nova Scotia) [19,33,80]; (b) media (e.g., BBC’s Blue Planet II; National Geographic’s “Planet or Plastic”) and apps (e.g., The Marine Debris Tracker, SeaCleaner) [81,82]; (c) beach clean-ups, useful in awareness and remediation (e.g., Great Canadian Shore Cleanup) [83,84]; (d) and inexpensive but valuable citizen science that could help map marine litter [79,85].

**Figure 2:**
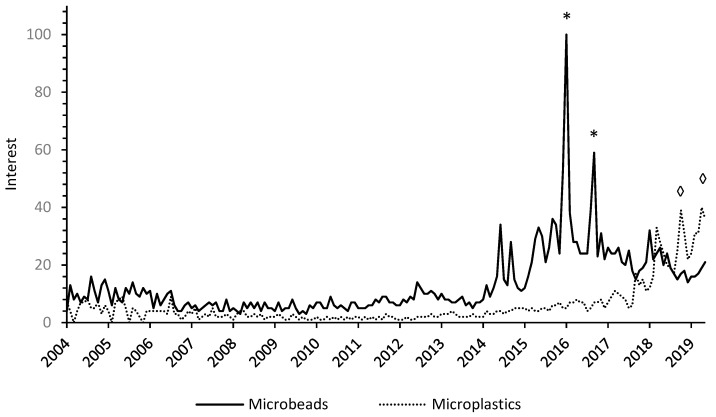
Google search volume for “Microbeads” and “Microplastics” simultaneously for the period of January 2004 to May 2019, retrieved in May 2019 from Google Trends and presented in interest (obtained by dividing the number of searches by the total amount of searches in the presented data). The highest peaks are related to social media articles with higher engagement (e.g., shares, likes, interactions) for those years, namely news articles, obtained from Buzzsumo (app.buzzsumo.com/research/most-shared). The two highest peaks for “microbeads” happened in 2016 (*) in May, related to “Ban Microbeads” by Greenpeace, and in September, related to “The UK will ban plastic microbeads by 2017” by IFL Science. For “microplastics,” the highest peaks (◊) happened in October 2018, related to the news articles “Microplastics found in 90 percent of table salt” by National Geographic, and in April 2019, related to “Microplastics end up in creatures in the deepest parts of the ocean” by Business insider.

The internet can be exploited as an education tool as 80% of search queries aim at retrieving information [86,87] whereas social media creates opportunities to engage with information [86,88], now having a greater impact than other information outlets [89]. When exploring the trends on the keywords “microplastics” and “microbeads” on search engines and social media, the evolution of the increased recognition of “microplastics” by the public was observed, as well as the behavior pattern consisting of consecutive release of news by media, sharing by social media, and triggering of information seeking behaviors on search engines, leading to an alternative route of active learning and awareness on this environmental issue (Figure 2).

Moreover, education and awareness campaigns should be successfully translated into long-term behavior changes [29,67], for instance, by reducing littering through moral obligations instead of ineffective littering sanctions [90,91]. Thus, education and awareness must focus on practical actions, including the reduction in consumption of harmful products, reduction in littering, and improving recycling rates, which could be delivered taking advantages of consumer’s online behavior. Nonetheless, reduction of plastic consumption is dependent on the availability of plastic-free alternatives [67].

## 4. Improving the Disposal of Waste

Waste management is based on the hierarchy of the four R’s: Reduce, Reuse, Recycle, and Recover. Even though the priority is to reduce and reuse, intervening in production and consumption, some waste will be produced and should be properly managed as a resource [1] through a suitable Integrated Waste Management System [34]. Reusing packaging is difficult, requiring recovery, sorting, and refill of packaging, and so it is scarcely used outside high-valued goods, such as electronics and vehicles [37]. Thus, when waste is produced it should be recycled, and only when it is not recyclable used as feedstock or in energy recovery, and only final waste, such as ash, landfilled. Proper solid waste management will reduce plastics in the environment, and therefore, decreases the fragmentation into microplastics [92]. For example, in Taiwan, improvement in waste management policies, such as bans of plastic bags and plastic tableware (the “Plastic Restriction Policy”) and mandatory sorting of waste (Recycling Act and Compulsory Trash-sorting Policy) were effective in reducing waste disposal rate (from 0.9 to 0.48 kg capita^−1^) and significantly reduced plastic bags, plastic bottles, and metal beverage cans in beaches [16]. Likewise, councils in Australia with higher budget investment in waste management had less litter on their coast [93]. Furthermore, regulations are now in place for the trade of mixed plastic scrap between countries, as agreed in the latest Basel Convention signed by over 180 countries [94], reducing the ability to export plastic waste and increasing the need for local solutions.

However, implementation of Integrated Waste Management Systems is expensive and slow. Developing countries lacking waste management may not be able to implement such complex systems right away. In these cases, containing waste is a priority to avoid public health risks and the production of marine litter. For this reason, landfills and incinerators may be used as the main strategies to manage waste, hopefully developing into more sustainable practices. Marine litter is a global problem requiring global action and cooperation. Thus, international cooperation may also aid this transition by providing knowledge or subsidizing waste management infra-structures in poor countries.

### 4.1. Disposal and Collection of Waste

The first step is the collection of waste by source-collection (by consumers) or post-separation (in centers) [95]. Source-collection is preferred because it is cheaper and reduces contamination of waste. Disposal of waste, from more to less convenient for consumers (and inversely for municipalities responsible for collection), include (a) door-to-door collection, with or without fees; (b) curbside collection; and (c) buy-back centers (buying litter) or drop-off centers (not buying litter) [37,95]. Economic incentives to increase recycling rates can be positive, such in buy-back programs where a sum of money is received (or returned) to the consumer per package or weight, or negative, in the case of fees varying with weight and waste type (with lower fees for recyclables) in door-to-door collection [96] or by using smart trash containers. However, setting fee values is a delicate business: high fees may cause illegal dumping or burning of waste whereas low fees will not affect consumption and separation of waste [46]. On the contrary, buy-back programs reduce littering, illegal dumping, and costs of collection. For example, the number of beverage containers in the coast of the United States and Australia decreased after the implementation of container deposit legislation, a buy-back program [97]. A cost-benefit analysis in Israel of deposit-refund programs for beverage containers demonstrated the economic benefits of this strategy, mostly from avoiding the transportation of high volume-low weight beverage containers by curbside waste collection [98]. Even though this collection strategy is related to increased recycling rates and reduced littering, it is also criticized due to its high costs, increased environmental impact by maintaining deposit-refund systems and curbside collection in parallel, and the high recycling rates in some countries despite not applying this strategy [99]. Thus, the use of deposit-refund or buy-back programs must be individually evaluated for each region and material.

Door-to-door collection with fees also has potential to reduce waste per capita and improve recycling participation. Even though this method is expensive for municipalities, costs may be lower than landfilling and recovering waste from the environment and it follows the “pay-as-you-throw” philosophy. Such door-to-door programs are already successfully implemented in places like Germany and San Francisco, USA. Nonetheless, door-to-door systems require citizens to store waste in their homes, threatening public health and privacy, and have high environmental impacts resulting from longer waste collection routes conducted by fossil-fueled vehicles [100]. As an alternative, smart trash containers, opened by resident cards and allowing only the deposition of a limited waste volume in each opening, could provide curbside collection of waste and rightful application of waste fees depending on the volume produced by each household (pay-as-you-throw). Increased number of waste containers in curbside collection, as well as increased variety of containers for source separation, may improve recyclability and waste disposal, but must take into account the increase in collection efforts by fossil-fueled vehicles and the decreased mass of single waste streams that may raise recycling costs [101].

#### Avoiding littering

Incorrect disposal of waste, known as littering, is also a contributor to marine litter. Until now, sanctions have been applied as a dissuasive method for littering. However, sanctions are ineffective because they require constant monitoring [91]. Thus, they could be coupled with positive reinforcements, such as offering incentives for the correct disposal of waste [91], or by appealing to the moral obligations of the individual, since behaviors can be reduced by social sanctions such as shame (self-imposed) or embarrassment (socially imposed) [90]. Positive reinforcements include tax incentives or deposit-refund systems, that may also be used to improve recycling rates [36,95].

### 4.2. Recycling

Recycling of plastics is a complex process comprised of (1) collection of separated waste by consumers or in centers; (2) separation of recyclables and elimination of contaminants; (3) grounding and segregation by polymer and color (e.g., by Fourier-transformed near-infrared spectroscopy); (4) extrusion of each polymer and color in pellets; and (5) selling recycled pellets to manufacturing companies [34,39,48,95,102]. Separation of waste by polymer is especially difficult and may compromise the final quality of recycled plastics.

Primary recycling (closed-loop) generates high quality plastics from uncontaminated materials usually produced by manufacturers (e.g., plastic back covers on flat screen televisions), whereas secondary recycling (downgrading) generates lower quality plastic to be used in less demanding applications (e.g., construction materials, textiles, asphalt, concrete, and composites) from contaminated plastics, such as consumer waste [39,88,103,104,105,106]. Ideally, recycled plastics should be used in long life and durable applications [39,102]. Shredded plastics waste can also be incorporated in asphalt [106] and concrete [103] to improve their properties. By using a cross-linking compatibilizer agent, mixed polymers or mixtures of plastics and non-plastics (e.g., wood) can also produce durable and low-cost thermoset composites [39,102,104] that can be used, for instance, as railway sleepers [107]. Composites may reduce the need for separation by polymer and are more durable for outdoor use than their conventional counterparts (e.g., wood) but cannot be further recycled.

Recycling plastics, especially originating from consumer waste, still is limited by (a) high cost of recycling process compared to the low-cost of virgin plastics [33]; (b) degradation and contamination of plastics that limits its uses and number of recycling cycles [39,108]; and (c) low recyclability of some plastic products such as textiles, flexible packaging, or laminated plastics [37,102]. Recycling of plastics is usually not economically feasible but may generate economic returns in closed-loop systems [109,110]. Conversely, manufacturers need a constant supply of raw materials of standard quality—sometimes hard to achieve with recycled plastic. We can hope that by increasing recycling rates, improving quality of recycled materials, and technological developments in the recycling process may overcome these problems.

Considering the environmental impacts, recycling is the preferred method of waste management [110,111,112,113,114]. For example, recycling PET and PE only require half the energy necessary to produce virgin polymers [111]. However, high organic contamination and low ability to replace virgin plastics may favor incineration over recycling [115]. Recycling impacts on the environment usually result from the consumption of non-renewable energy, transportation, and introduction of fillers and additives [109,111,112,116].

Nonetheless, recycling is still a priority in waste management. Recycling saves resources and energy, reduces pollutant emissions, reduces the need for landfills, creates jobs and improves local economies, reduces imports of resources, and generally improves the environment. Therefore, even though recycling is expensive, it benefits society and is less expensive than the alternatives. For these reasons, countries should try to improve their recycling rates. Recycling may also become economically feasible by improving primary recycling, imposing taxes on raw plastics or through the mandatory use of recycled plastics in all products, as previously discussed. Nonetheless, not all plastics can be recycled—mixed, contaminated, and degraded plastics are not suited for recycling but can be used as feedstocks or in energy recovery.

### 4.3. Feedstock and Waste-to-Energy

Waste-to-energy is the production of steam, heat, electricity, or fuel from waste. Waste-to-energy strategies for plastic allow to recover energy and overcome recycling limitations such as the need for sorting by polymer and competition with virgin plastics [117]. However, it perpetuates a linear economy.

Reformulation of plastics into feedstocks uses energy to recover constituents that can be used to produce fuels similar to gasoline (waste-to-energy) or to produce chemicals, lubricants, and carbon black [34,37,106,118]. Pyrolysis, an endothermic cracking process without oxidation, and gasification, a similar process with partial oxidation [38], are efficient in producing fuels from plastic waste with similar physicochemical properties and costs as gasoline, especially when produced in the presence of catalysts [118]. Besides fuel production, valorization may come from by-products, such as using carbon black to improve asphalt [106], or by producing value-added substances, such as carbon nanotubes produced from plastic waste at a lower cost and with lower CO_2_ emissions [119]. On-board gasification of plastic marine litter has even been proposed as an energy source for boats [120].

Conversely, oxidation of plastics (incineration), considered high calorific waste (43.3 MJ·kg^−1^ for polyethylene vs. 42.5 MJ·kg^−1^ in heavy fuel oil), produces steam, heat, or electricity allowing energy recovery, reduction of waste volume, and elimination of harmful substances or organisms [34,35,102,118,121]. Contaminated or mixed residues that cannot be recycled may be diverted to incineration [35,102]. Plastic waste can be used as an energy source in cement furnaces [121], chemical waste incinerators, metal melting ovens [102], and electric arc furnaces in steelmaking [118]. The resultant inert ash can be deposited in landfills or used in metal recovery programs [35] or aggregates in road construction [102,106].

Thus, benefits from waste-to-energy include (a) higher energy savings than recycling [110]; (b) not requiring pre-treatment of waste; (c) treatment of mixed or contaminated wastes; (d) neutral or slightly positive environmental impact depending on efficiency [112]; and (e) replacement of fossil fuels, especially in high efficiency and electricity to heat ratios [99]. Conversely, limitations of incineration include (a) long-term investment [35]; (b) release of dangerous substances into the atmosphere [34,37], which may be reduced by using a second combustion chamber [39]; and (c) perpetuation of the linear economy [102]. Incineration requires expensive and advanced air pollution control, due to the heterogeneity of waste, the release of toxics from waste (e.g., monomers or additives from plastics), and the release of high amounts of CO_2_ to the atmosphere; nonetheless these limitations could be partially overcome by the use of high efficiency and high electricity-to-heat ratio when wastes replace fossil fuels in energy production [99]. Furthermore, incineration results in the production of two types of ash (bottom ash and fly ash) that contain a high concentration of contaminants (e.g., heavy metals, salt, chloride, organic pollutants) [99,122]. Alternatives to the landfilling of ash, the most common method of disposal due to the presence of hazardous contaminants, include its use in applications (e.g., concrete pollution, road pavement, and glasses and ceramics) after expensive pre-treatment techniques [99] or the use in metal recovery programs (e.g., through solvents), allowing the concentration and reintroduction of these metals in the circular economy [122]. Eriksen et al. [123] argue that gasification may also overcome some of these problems, producing energy at a cheaper price, with short-term returns and higher conversion efficiencies, acting as a transition to more sustainable waste strategies. Thus, feedstock and waste-to-energy are important short-term strategies, diverting litter from landfills, but will progressively decrease in interest as recycling programs are improved. Even though recycling rates should increase, there will always be fractions of refuse comprised of mixed, contaminated, or degraded plastics that will be suited for these operations.

### 4.4. Landfilling

Landfills are part of a linear waste management but also take part in an integrated management as end-of-life residues are produced by recycling and incineration [38]. Nonetheless, they should always be the last option because of higher environmental impact, higher risk of contamination, loss of resources, and land requirements [37]. Thus, landfills should only be used as a last resort, to accommodate wastes resulting from recycling, production of feedstock, or waste-to-energy. Furthermore, landfill mining is seen as alternative to the re-introduction of landfilled wastes in the circular economy, either by recycling (e.g., metals) or through waste-to-energy (e.g., plastics) [124]. Indeed, the amount of plastics buried in landfills in China could produce 38% of its total annual energy consumption if reclaimed [125]. Waste-to-energy could be part of clean-up projects (i.e., of reclaimed landfill plastics) and integrated waste management mainly through implementation of regional gasification facilities, with calorific values evaluated through thermogravimetric analysis [124]. Nonetheless, this technology is still limited by the different thermal behavior of waste, high heterogeneity, moisture and impurities content, as well as the production of ash [124].

### 4.5. Bio-Based and Biodegradable Plastics as Alternatives to Conventional Plastics

The increasing importance of sustainability has boosted the search for green materials, aiming at replacement of fossil fuels with renewable resources and increase in recycling targets and waste management efficiency [126,127,128,129]. The terms bio-based, degradable, and biodegradable are often misinterpreted and improperly used [128]. Bio-based polymers (or bioplastics) are polymers derived from renewable feedstocks (i.e., from biomass), independent of biodegradability (e.g., bio-polyethylene) [130,131,132]. Degradable polymers contain additives or polymers that will break down when triggered by composting, in the case of biodegradable plastics, or by exposure to UV radiation, in the case of photochemically degradable plastics, which often produce non-degradable microplastics [48,133]. Independent of their feedstock origin, biodegradable polymers undergo processes of degradation into water, carbon dioxide, and organic matter (mineralization) with the action of naturally occurring microorganisms (e.g., bacteria, fungi, or algae), but may require conditions (e.g., temperature) not present under environmental conditions (e.g., polylactic acid, PLA) [134,135,136]. An alternative to the recycling and composting is waste-to-energy, with the incineration of bio-based plastics being carbon-neutral, producing renewable energy [135,137]. Moreover, conventional plastics may biodegrade when exposed to specific plastic-degrading organisms (e.g., *Zalerion maritimum*), producing valuable biologic products or organic matter [138], offering an alternative for contaminated or degraded plastics.

Biodegradable polymers offer solutions for short life-span applications, at risk of entering the environment, or where composting is desirable, such as agricultural films, packaging, and disposable cutlery [128,137]. However, a lot of uncertainties regarding biodegradable polymers remain, including (a) the increasing complexity in waste management, including the need for specific collection and composting facilities, the low volumes produced not justifying waste management efforts, the degradation of common plastics if introduced in recycling lines, and longer degradation periods and release of greenhouse gases under anaerobic conditions in landfills (likely in developing countries); (b) the presence of contaminants that may compromise compost quality or release harmful chemicals or particles to the environment, such as additives used to enhance physical properties; (c) the high costs of production coupled with ensuring the lack of degradation before use, which may compromise product shelf-life; (d) the use of agricultural land and chemical fertilizers, insecticides, and herbicides, in the case of bio-based polymers; (e) increased littering due to biodegradable claims coupled, in some cases, with low rates of biodegradation in the environment [1,48,128,135,137,139,140,141,142,143,144,145,146,147]. For example, in Thailand, LCA of water bottles reveals that cassava-based PLA bottles generally have lower environmental impacts than its fuel-based PET, with the exception of eutrophication and acidification potential caused by agricultural processes [148], whereas the PLA food trays have higher environmental impacts than PS [149]. Hence, the same material can have higher or lower impacts depending on the production, process, and waste management strategies, which can be clarified by LCA analysis.

Even though biodegradable plastics currently increase the complexity of waste management, their shortcomings and impacts are likely to decrease in the future as sustainable solutions are found. Moreover, non-degradable plastics could be a part of the circular economy considering correct use and disposal, the use of current infrastructures, and reductions in greenhouse gas emissions when bio-based. As for other materials, LCA analysis can provide evidence for an informed decision.

### 4.6. E-Waste

E-waste (electronic and electrical waste) contains a complex mixture of materials and is a fast-growing segment of consumers waste. E-waste includes household appliances, information and communication technology, and consumer electronics [150]. Recycling of e-waste starts with shredding that results in separation of metals from the plastics [151], followed by separation of metals and finally, separation of plastics by the same methods described before in primary and secondary recycling [150]. However, recycling of plastics is compromised by the presence of contaminants, such as paint and brominated flame-retardants [151]. Replacement of plastic in printed circuit boards by biodegradable natural fibres or proteins recovered from agriculture waste or co-products, such as banana stems or wheat gluten, is possible [152]. Substitution, along with re-design of electronics to reduce the number of polymers used and increase recyclability, may help reduce the environmental impact of e-waste [151]. Until then, few plastics from e-waste are recyclable but most can be used in waste-to-energy recovery.

## 5. Recommendations

Proposed recommendations to reduce the loss of (micro)plastic to the environment during production, consumption and disposal (i.e., mismanagement), organized by their priority, include:

- Short-term measures:
  - Regulation of production and consumption through bans or taxes of plastic products that are harmful to the environment, without compromising public health or food safety;
  - Reducing the consumption of plastics through removal of unnecessary packaging (e.g., double packaging), labelling, awareness, education, and by providing eco-friendly alternatives to plastics when possible without unintended consequences;
  - Increasing demand for recycled plastics through benefits, sanctions, or taxes on virgin plastics;
- Mid-term measures:
  - Implementation of waste collection systems that lead to reductions in waste production, improve recycling rates following the “pay-as-you-throw” principle, such as door-to-door collection and deposit-refund systems;
  - Prioritizing recycling followed by feedstock and waste-to-energy that allow recovery of valuable chemicals and energy; landfill should only be used in waste produced in the previous processes;
  - Reduction and recycling of waste formed during production and responsibility over waste and impacts caused by products (EPR);
- Long-term measures:
  - Using renewable energy during collection of waste and recycling to reduce environmental impacts of recycled plastics;
  - Implementation of LCA for each product and process to improve eco-design (including reuse, repair, and recyclability), taking into consideration expected end-of-life of products;
  - Using bio-based plastics to reduce environmental impacts from fuel-based plastics; reducing production of degradable plastics that produce harmful fragments (microplastics); using biodegradable plastics in applications where composting is beneficial (e.g., agricultural films) while providing specific collection and waste treatment (since biodegradation may be slower or not possible in the environment);
  - Improving recyclability of e-waste and in the interim, disposal through waste-to-energy.

## 6. Conclusions

Misuse and mismanagement of durable plastics has led to large accumulations of this material in the environment (plastic pollution), posing a risk to organisms, ecosystems, and human health. This study discusses current knowledge on improvements to production, consumption, and disposal of plastic, providing stakeholders with 10 recommendations to reduce the loss of plastic to the environment during production, consumption, and disposal. The life-cycle of plastics must be improved through an integrated waste management system, reducing their environmental impacts and following the hierarchy of waste management using the four R’s: reduce, reuse, recycle, and recover. Thus, (micro)plastic production and consumption must decline by improving design or using alternatives materials (reduce) and creating durable products (reuse). Nonetheless, societies will produce waste. If improvements are made at the production stage, most plastic waste can be recycled, partially substituting virgin plastics in new products. Plastics that cannot be recycled can be recovered to produce chemical components (feedstock) or to produce energy (waste-to-energy). Only the waste produced by these activities should be landfilled. These measures require command-and-control and economic measures created by governments, voluntary measures from industries, and changes in consumer behavior. Since plastic marine litter knows no boundaries, international cooperation to improve waste management systems in all countries (or at least coastal countries) is required. As the concentration of plastics in the oceans stabilize, clean-up activities can remove plastics in the environment, sending them to waste management, helping ecosystems to recover from plastic pollution.
